# Reduced graphene oxide coating enhances osteogenic differentiation of human mesenchymal stem cells on Ti surfaces

**DOI:** 10.1186/s40824-021-00205-x

**Published:** 2021-02-12

**Authors:** Moon Sung Kang, Seung Jo Jeong, Seok Hyun Lee, Bongju Kim, Suck Won Hong, Jong Ho Lee, Dong-Wook Han

**Affiliations:** 1 Department of Cogno-Mechatronics Engineering, College of Nanoscience & Nanotechnology, Pusan National University, Busan, 46241 South Korea; 2 GS Medical Co., Ltd., Cheongju-si, Chungcheongbuk-do 28161 South Korea; 3 Department of Optics and Mechatronics Engineering, College of Nanoscience & Nanotechnology, Pusan National University, Busan, 46241 South Korea; 4 Dental Life Science Research Institute / Innovation Research & Support Center for Dental Science, Seoul National University Dental Hospital, Seoul, 03080 South Korea; 5 Daan Korea Corporation, Seoul, 06252 South Korea

**Keywords:** Titanium, Reduced graphene oxide, Osteogenesis, Bone tissue engineering, Surface coating

## Abstract

**Background:**

Titanium (Ti) has been utilized as hard tissue replacement owing to its superior mechanical and bioinert property, however, lack in tissue compatibility and biofunctionality has limited its clinical use. Reduced graphene oxide (rGO) is one of the graphene derivatives that possess extraordinary biofunctionality and are known to induce osseointegration in vitro and in vivo. In this study, rGO was uniformly coated by meniscus-dragging deposition (MDD) technique to fabricate rGO-Ti substrate for orthopedic and dental implant application.

**Methods:**

The physicochemical characteristics of rGO-coated Ti (rGO-Ti) substrates were evaluated by atomic force microscopy, water contact angle, and Raman spectroscopy. Furthermore, human mesenchymal stem cells (hMSCs) were cultured on the rGO-Ti substrate, and then their cellular behaviors such as growth and osteogenic differentiation were determined by a cell counting kit-8 assay, alkaline phosphatase (ALP) activity assay, and alizarin red S staining.

**Results:**

rGO was coated uniformly on Ti substrates by MDD process, which allowed a decrease in the surface roughness and contact angle of Ti substrates. While rGO-Ti substrates significantly increased cell proliferation after 7 days of incubation, they significantly promoted ALP activity and matrix mineralization, which are early and late differentiation markers, respectively.

**Conclusion:**

It is suggested that rGO-Ti substrates can be effectively utilized as dental and orthopedic bone substitutes since these graphene derivatives have potent effects on stimulating the osteogenic differentiation of hMSCs and showed superior bioactivity and osteogenic potential.

## Introduction

Most tissues in the body regenerate over a lifetime, but damage beyond a recoverable range or certain tissues are often irreversibly damaged by internal or external factors such as traumatic injuries, cancers, bacterial or viral infection, and degenerative disease that leads to functional and cosmetic defects. To overcome the issues, tissue engineering has emerged as a promising approach to treat the loss or defectives, thereby improve the wound healing process. Recently, many tissue engineering studies have focused on the development of artificial scaffolds to mimic the structural and functional characteristics of natural extracellular matrix (ECM) [1–5]. Recently developed artificial scaffolds aim to reproduce the physicochemical and mechanical property of natural ECM because the destiny of cells is highly affected by the surrounding microenvironment. Several biofunctional cues such as nanomaterials, biomolecules, and drugs have been incorporated into the scaffold to regulate the cellular behaviors and maintain the intrinsic properties of cells [6–10]. Titanium (Ti) has been utilized as hard tissue replacement such as dentistry, bone, and joint substitutes due to the light but strong mechanical property, durability, non-immune reaction, and non-degradability [11, 12]. Generally, successful replacement requires hard tissue compatibility for new bone formation and osseointegration, and soft tissue compatibility for epithelial adhesion [13, 14]. However, Ti has no enough biofunctionality, leading to low interaction with original tissues, and inhibits the adsorption of proteins and cell adhesion. To overcome this issue, surface coating and treatment have been introduced to change the composition, chemical reactivity, and morphology of the Ti surface while maintaining the suitable mechanical properties of Ti itself [15–19].

On the other hand, graphene is one of the novel nanomaterial family, which is composed of two-dimensional monolayered sp^2^-bonded carbon atoms and features exceptional physicochemical, electrical, and mechanical properties. Graphene and their derivative have been explored increasingly for biomedical applications including drug delivery carriers, imaging probes, biosensors, and tissue engineering scaffolds [20–24]. Graphene is obtained by physicochemical exfoliation of graphite, whereas its derivatives such as graphene oxide (GO), a highly oxidative form of graphene, and reduced GO (rGO), which is prepared by chemical or thermal reduction of GO, have their specific characteristics. The extraordinary biocompatibility of graphene derivatives enables wide application in biomedical fields [25].

In particular, graphene and its derivatives can be utilized as tissue engineering scaffold materials because they have known to enhance cellular behaviors such as adhesion, proliferation, and migration [26–28]. Owing to the hydrophilic and cell-adhesive nature, graphene and its derivatives are utilized as the micro-patterned scaffold to enable contact guidance of cells [29–32]. Furthermore, previous researches indicated that graphene derivatives induce cells to differentiate to specific lineages such as adipogenesis, chondrogenesis, myogenesis, neuritogenesis, and osteogenesis [33–40]. Especially, graphene derivatives such as GO and rGO are known to enhance protein adsorption and cell-cell or cell-matrix interaction, hence, support osteogenic differentiation of pre-osteoblasts and stem cells [41–43]. Compared to GO, rGO possesses structural defects to enhance the interactions with biomolecules, cell, and polymers. Furthermore, by controlling the C/O ratio, electrical conductivity and hydrophilicity of the rGO can be precisely tailored to induce optimal cell-matrix interaction responsible for modulating kinds of biological processes [44].

The extraordinary osteogenesis-inducing capability of GO and rGO made them applied as bone tissue engineering scaffold collaborating with kinds of biomaterials such as hydroxyapatite, gelatin hydrogel, calcium phosphate, and RGD peptide [45–49].

In this study, rGO-coated Ti (rGO-Ti) substrates were fabricated as in vitro culture platform of human mesenchymal stem cells (hMSCs) and to explore their potential as artificial scaffolds for bone tissue engineering. The physicochemical properties of fabricated scaffolds were analyzed by atomic force microscopy (AFM), contact angle measurements, and Raman spectroscopy. Subsequently, the cellular behaviors such as proliferation and osteogenic differentiation of hMSCs on the prepared scaffolds were evaluated.

## Methods

### Preparation of rGO nanoparticles (NPs) and rGO-Ti substrate

4000 ppm GO solution was purchased from Sigma-Aldrich (St. Louis, MO). For the reduction process as described elsewhere [28, 46], GO (1 g) was sonicated in 1 L deionized water for 2 h. Hydrazine hydrate (10 mL) was then added to the suspension and the reaction proceeded at 100 °C for 24 h. After the reaction, the suspension was filtered and washed several times with water/ethanol solution. Finally, the rGO NPs were prepared after drying in a vacuum oven at 80 °C for 12 h.

**Fig. 1:**
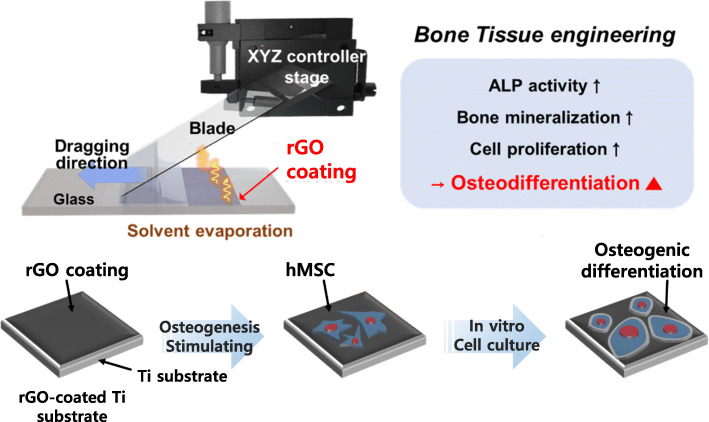
Schematic diagram of meniscus-dragging deposition technique and the osteodifferentiation-inducing capability of the rGO-Ti substrate

As shown in Fig. 1, rapid film deposition was achieved using the meniscus-dragging deposition (MDD) technique [42]. The Ti substrates were used as a deposition plate and a coating plate. They were cleaned with a piranha solution for 30 min and rinsed with deionized water. This is a short time process; the deposition plate was placed on the coating substrate at an angle of 30° and a 50 mL droplet of the rGO solution was injected into the wedge between the two plates. The deposition plate was pushed linearly in an alternating back-and-forth motion (one alternating motion is defined as one deposition number) by a motorized stage (AL1–1515–3S, Micro Motion Technology, Valley Center, USA) at a constant speed of 250 mm/s to deposit the rGO on the coating substrate. The coating area of the rGO-Ti membranes was 5 × 5 cm^2^. The large-area rGO thin films on the Ti substrate were coated with a rGO concentration of 100 μg/mL. Subsequently, the rGO-Ti membranes were dried at 80 °C for 30 min.

### Characterizations of intact Ti and rGO-Ti substrate

Intact Ti and rGO-Ti substrates were washed with acetone and DPBS three times respectively and dried at RT for every experiment in this section. Intact Ti and rGO-Ti substrates were imaged by AFM (XE-100, Park Systems) with a silicon cantilever in non-contact mode. The images with 10 μm × 10 μm scan sizes consisted of 512 × 512 points of height data and a scan rate of 1.0 Hz/line. R_q_ represents the root mean square value of the height raised from the standard when filling a lower valley from a height higher than the standard. R_q_ values were calculated with three separate samples at various location as follow:

$${R}_q=\sqrt{\frac{1}{l}{\int}_0^l{y}^2(x) dx}$$

Raman spectra of intact Ti and rGO-Ti substrates were recorded using a Raman spectroscopy (Ramboss 500i, Dong Woo Optron Co., Gwangju, Korea) equipped with a charge-coupled device camera (iDusDV420A-OE, Andor Technology, Belfast, Ireland) and a precise motorized stage (SGSP 20–85, Sigma Koki Co., Ltd., Tokyo, Japan). Ar-ion laser of 532 nm (LasNova 50, LASOS, Jena, Germany) was focused on the sample using a water immersion objective lens (× 60 magnification, the numerical aperture of 1.2 UPlanSApo, Olympus, Tokyo, Japan) and resolved with a monochromator (Monora500i, DongWoo Optron Co., Gwangju, Korea). 5 mW laser power at 532 nm that is attenuated by using a neutral density filter of 50% transmittance at the objective was used at a range of 1300–2900 cm^− 1^. Water contact angles of the intact Ti and rGO-Ti substrates were measured the sessile drop method using the OCA 10 goniometer (Dataphysics, Filderstadt, Germany). The 10 μL of distilled water was dropped onto the surface of the intact Ti and rGO-Ti substrates and the images were captured, thereafter, the contact angle was calculated by an optical system.

### Cell culture and conditions

hMSCs were purchased from Lonza (Walkersville, MD, USA), and all experiments were conducted using hMSCs between passages 3 and 5. To maintain undifferentiated state, hMSCs were routinely cultured in MSC basal medium (Lonza) containing 10% of MSC growth supplement (Lonza), 2% of l-glutamine, 0.1% of GA-1000, and 1% of antibiotic-antimycotic solution (10,000 units of penicillin, 10 mg of streptomycin, and 25 μg of amphotericin B per mL, Sigma-Aldrich) at 37 °C under 5% CO_2_ in a humidified atmosphere. For osteogenic differentiation assay, hMSCs were cultured in Dulbecco’s modified Eagle’s medium (DMEM) supplemented with 10^− 8^ M dexamethasone (Abcam), 0.2 mM ascorbic acid (Sigma-Aldrich), and 10 mM β-glycerolphosphate (Sigma-Aldrich).

### Cell proliferation assay

To evaluate the proliferation of hMSCs on intact Ti and rGO-Ti substrates, a cell counting kit-8 (CCK-8) assay (Dojindo Laboratories, Kumamoto, Japan) was performed according to the manufacturer’s protocol. hMSCs were seeded at a density of 1 × 10^4^ cells/mL on 10 × 10  mm^2^ intact Ti and rGO-Ti substrate, and cultured at 37 °C under 5% CO_2_ in a humidified atmosphere. After 1, 7, 14, and 21 days of incubation, each sample was washed twice with DPBS solution, and then cultured with a CCK-8 solution for 2 h at 37 °C in the dark under 5% CO_2_ in a humidified atmosphere. The absorbance at 450 nm was assessed at each time point by using a SpectraMax® 340 plate reader (Molecular Devices Co., Sunnyvale, CA, USA).

### Alkaline phosphatase (ALP) activity assay and alizarin red S (ARS) staining

To investigate the osteogenic differentiation of hMSCs, ALP activity assay was conducted. The hMSCs were seeded at a concentration of 1 × 10^4^ cells/mL on intact Ti substrate and rGO-Ti substrate and incubated for 1, 7, 14, and 21 days. ALP activity of hMSCs was determined by measuring the conversion of ρ-nitrophenyl-phosphate to ρ-nitrophenol by an ALP assay kit (Abcam, Cambridge, UK) as per the manufacturer’s protocol. The absorbance at 405 nm was assessed at each time point by using a SpectraMax® 340 plate reader. ALP activity was estimated by calculating the total amount of ρ-nitrophenol formation (μmol) divided by the reaction time (min) and volume of sample (mL) (OD value*1000/60/0.24).

To monitor the extracellular calcium deposits of hMSCs, the cells were seeded at a concentration of 1 × 10^4^ cells/mL on intact Ti substrate and rGO-Ti substrate and incubated for 1, 7, 14, and 21 days. At each time point, hMSCs were washed twice with DPBS solution, fixed with 3.7% of formaldehyde for 10 min, and stained with 40 mM of ARS in DPBS solution (pH 4.2, Sigma-Aldrich). The hMSC-cultured substrates were imaged with a digital camera (Olympus Optical Co., Tokyo, Japan). Quantitative analysis was performed by extracting ARS in stained hMSCs. To extract ARS from stained hMSCs, 10% of acetic acid solution was added, and hMSC-cultured substrates were incubated for 30 min with constant shaking at 80 rpm. After then, 10% of ammonium hydroxide solution was added to neutralize the aqueous solution of the ARS extracts, and the absorbance values were measured using a SpectraMax® 340 plate reader at 405 nm.

### Statistical analysis

All variables were tested in three independent cultures for each experiment, which was repeated twice (*n* = 6). The quantitative data are expressed as the mean ± standard deviation (SD). The data were tested for the homogeneity of the variances using the Levene test, before statistical analysis. Statistical comparisons were carried out using a one-way analysis of variance (ANOVA; SAS Institute Inc., Cary, NC), followed by a Bonferroni test for multiple comparisons.

## Results

### Physicochemical properties of intact Ti and rGO-Ti substrates

**Fig. 2:**
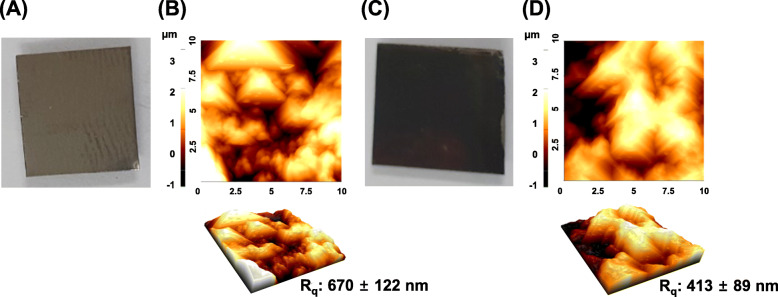
Surface characteristics of intact Ti and rGO-Ti substrates. Digital images of **a** intact Ti and **c** rGO-Ti substrates. AFM images of **b** intact Ti and **d** rGO-Ti substrates

Surface characteristics of prepared intact Ti and rGO-Ti substrates were characterized. As shown in Fig. 2a and c, the rGO-Ti substrates have darker surface than intact Ti showing rGO was uniformly coated on the Ti surface. Figure 2b and d indicate the representative AFM images and height profile of intact Ti and rGO-Ti substrates. Both substrates have a microscopically rough surface with a height ≤ 4 μm. In particular, the R_q_ value of rGO-Ti is relatively lower than that of intact Ti indicating the surface of rGO-Ti substrates with micro-scale grooves was partially flattened after nano-scale rGO NPs had been coated on them.

**Fig. 3:**
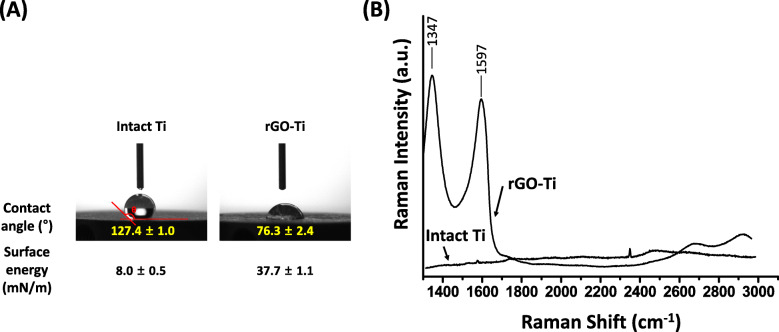
Physicochemical properties of intact Ti and rGO-Ti substrate. **a** Contact angle and **b** Raman spectra of intact Ti and rGO-Ti substrates

Fig. 3 represents the physicochemical property of intact Ti and rGO-Ti substrates. Water contact angle measurements (Fig. 3a) indicate the contact angle and surface energy of intact Ti and rGO-Ti substrate. Contact angle and surface energy of rGO-Ti was 76.3° ± 2.4° and 37.7 ± 1.1 mN/m, while that of intact Ti was 127.4° ± 1.0° and 8.0 ± 0.5 mN/m, respectively. The result indicated that rGO-Ti substrates are more hydrophilic than intact Ti substrates. However, it is a specific characteristic of rGO to have structural defects and retain residual oxygen moieties such as hydroxyl groups, carbonyl groups, carboxylic groups, and epoxide from graphene oxide. In other words, by maintaining the hydrophobic property of intact graphene to some extent, rGO potentially encourages the cells to adhere to the surface. A previous study compared the water contact angle between GO and rGO, indicating GO is hydrophilic while rGO is very hydrophobic. rGO-Ti substrates of our results are not very hydrophobic (water contact angle of rGO is < 90°), which attribute to the highly smoothed surface morphology [50].

Raman spectra revealed that intact Ti substrates did not exhibit any specific Raman peak indicating that there are no impurities on the prepared sample (Fig. 3b). Whereas, rGO-Ti substrates exhibited strong Raman peaks at 1347 cm^− 1^ and 1597 cm^− 1^. These peaks are characteristic Raman spectra of carbon nanomaterials, D band (~ 1350 cm^− 1^), and G band (~ 1600 cm^-^^1^) [51, 52]. G band depicts the hybrid carbon from the graphene and D band derived from the structural defects from sp^2^ hybrid carbon [53, 54]. Results indicated that the Raman intensity ratio of D band and G band (I_D_/I_G_) was 1.11, corresponding general I_D_/I_G_ value of rGO which is larger than 1. Therefore, it is considered that rGO was successfully prepared and coated on Ti substrates.

### Proliferation of hMSCs on intact Ti and rGO-Ti substrates

**Fig. 4:**
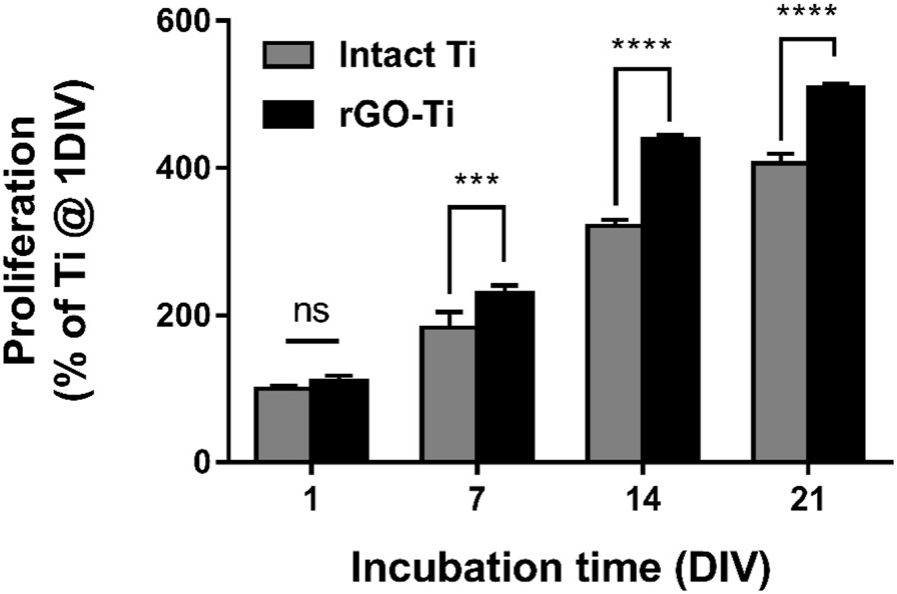
hMSCs proliferation on intact Ti and rGO-Ti substrates. The results are shown as the mean ± SD (*n* = 6, ns: not significant, ***: *p* < 0.001, ****: *p* < 0.0001)

The growth and proliferation of hMSCs on the bone-implant surface play a crucial role in osseointegration with original tissues. Thus the effect of rGO-Ti substrates on the growth of hMSCs was evaluated quantitatively. Fig. 4 shows the proliferation of hMSCs assessed by CCK-8 assay. After culturing hMSCs on intact Ti and rGO-Ti substrates, cell proliferation was assessed at days 1, 7, 14, and 21. Both intact Ti and rGO-Ti substrates showed a tendency to enhance cell proliferation. At 1 and 3 days of incubation, cell proliferation on rGO-Ti slightly increased compared to intact Ti substrates. On day 14 and 21, the proliferation of hMSCs on rGO-Ti substrates was significantly (*p* < 0.0001) increased compared to that of intact Ti substrates.

### ALP activity of hMSCs on intact Ti and rGO-Ti substrates

**Fig. 5:**
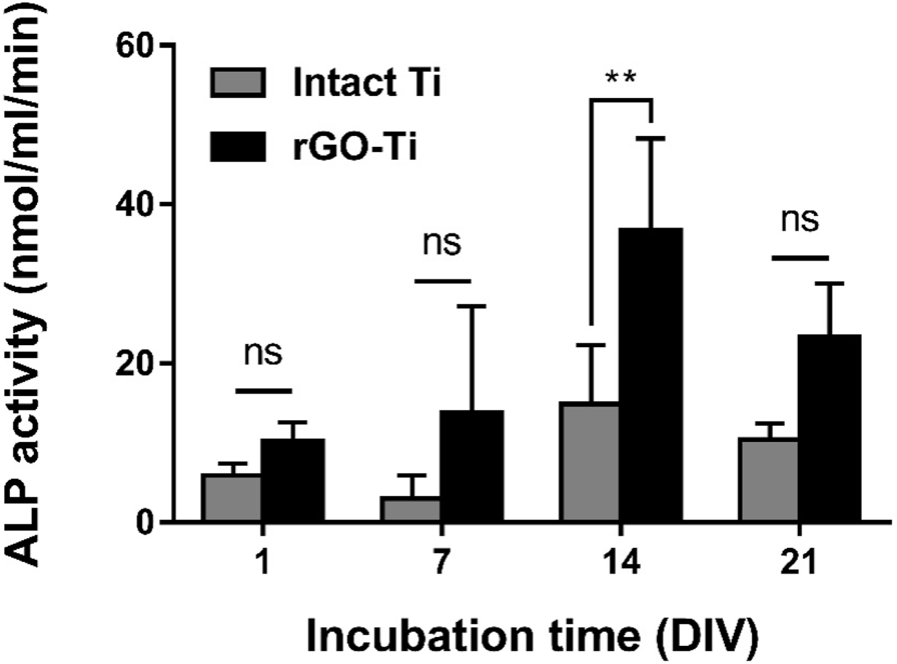
ALP activity of hMSCs on intact Ti and rGO-Ti substrates. The results are shown as the mean ± SD (*n* = 6, ns: not significant, **: *p* < 0.01)

Osteoblastic cells secrete ECM proteins and early differentiation markers such as ALP, which plays an important role in bone matrix deposition and mineralization by providing calcium and phosphate ions. To evaluate the osteogenic differentiation promoting the ability of Ti-rGO substrates, ALP activity of hMSCs was assessed. Figure 5 shows the ALP activity of hMSCs on intact Ti and rGO-Ti substrates after 1, 7, 14, and 21 days of incubation. Both intact Ti and rGO-Ti substrates showed the highest ALP activity at 14 days and tend to decrease at 21 days. ALP is one of the early markers of osteogenic differentiation, therefore, increases in the stage of osteogenesis and decreased when MSCs differentiate to osteocytes [55]. In particular, hMSCs showed significantly (*p* < 0.01) increased ALP activity at 14 and 21 days compared to that of intact Ti substrates. These results may be attributed to defective structure and the oxygen moieties of rGO which enhanced cell-matrix interaction and essential protein adsorption from serum.

### Mineralization of hMSCs on intact Ti and rGO-Ti substrates

**Fig. 6:**
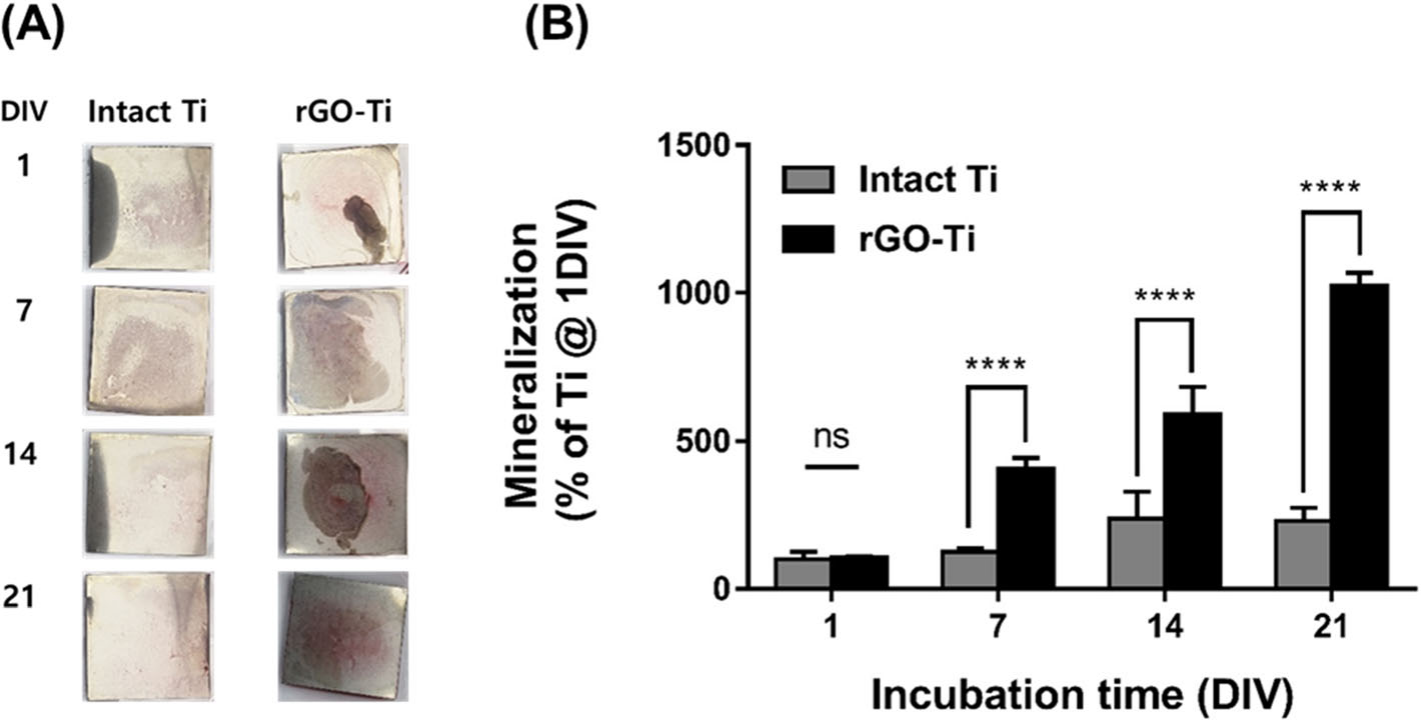
Mineralization of hMSCs on intact Ti and rGO-Ti substrates. **a** Digital images of ARS stained hMSCs on intact Ti and rGO-Ti substrates and **b** Quantification of ARS staining. The results are shown as the mean ± SD (*n* = 6, ns: not significant, ****: *p* < 0.0001)

To further explore the osteogenic differentiation-inducing effect of rGO-Ti, calcium phosphate deposition which is considered as a later marker for bone regeneration was observed by ARS staining. The image of ARS staining (Fig. 6a) and its corresponding graph (Fig. 6b) showed that rGO-Ti substrates significantly (*p* < 0.0001) increased extracellular calcium deposition in hMSCs, which was stained in red. While there was no significant difference between intact Ti and rGO-Ti substrates at 1 day, the notable formation of calcium deposits was observed on rGO-Ti substrates from 7 to 21 days. These findings suggest that rGO can induce an osteoid matrix deposition even without any osteogenic inducing agents.

## Discussion

In the present study, it was investigated whether rGO coating on Ti substrates can enhance the osteogenic differentiation of hMSCs without hindering cell growth for potential applications to bone tissue engineering and regeneration. To testify this hypothesis, the rGO-Ti substrates were prepared by the MDD process, which coats the intact surface of Ti substrates uniformly. After rGO coating, it was found that the Ti substrates showed a decrease in their surface roughness and water contact angle. Many studies have emphasized that surface roughness influences cell adhesion and regulates integrin-mediated signal cascades [56–58]. Moreover, rough surface enhances biomolecules adsorption, which can promote osteoblastic differentiation of hMSCs. A previous study reported the effects of Ti substrates with a different surface roughness on osteogenesis of SaOS-2 osteoblast-like cells [59]. SaOS-2 cells showed increased ECM protein synthesis and integrin protein expression on a rough surface, indicating accelerated cell-matrix adhesion according to surface roughness. Consequently, ALP activity was highly increased on the rough surface while there was no significant increase on the smooth surface [59]. However, the correlation of surface roughness and superficial morphology to enhanced osteogenesis of hMSCs is still controversial. Another study compared cell growth and expression of osteogenic markers of MG63 osteoblast-like cells according to the different surface roughness. Several markers such as ALP level, osteocalcin, procollagen type I, Transforming growth factor ß1 (TGF ß1), and nitrogen oxide level showed unrelated tendency according to the surface roughness [60]. These results suggest that various cell signaling are concerning osteogenesis, therefore, which means that it needs comprehensive correlation between the mechanisms of bone formation at the implant surface, the effects of the material on the surrounding cells and the profile of cytokines, growth factors, and other local mediators [61]. On the other hand, the wetting performance of the scaffold is critically important in bone tissue engineering because it directly determines the performance of cellular behaviors such as attachment, spreading, proliferation, and osteodifferentiation [62, 63].

For the cell study, the cell proliferation and osteogenic differentiation of hMSCs were examined on the rGO-Ti substrate. Even if the treatment concentration, cell type or culture conditions were different from this study, many related results demonstrated that rGO can induce positive effects on the proliferation of MSCs and preosteoblastic cells such as MC3T3-E1, hFOB, and MG63 cells [64–67]. This may be attributed to the osteogenic activity of rGO, which is known to promote cell adhesion, spreading, and proliferation by supporting the protein adsorption and intracellular protein delivery by the ionic bonding formation and the electrical conductivity [68, 69]. In particular, graphene has a high affinity to dexamethasone, β-glycerolphosphate, and ascorbic acid contained in osteogenic media which are well-known osteogenic inducers [70]. It is known that dexamethasone could upregulate many proteins and enzyme levels concerning osteogenesis, hence elaborate calcium deposition [71]. Meanwhile, dexamethasone synergistically acts with β-glycerolphosphate to enhance the ALP activity level in the cells and ascorbic acid favorably affects the maturation of osteoblasts [72]. The exceptional high affinity would be ascribed to the π–π stacking between aromatic rings of those biomolecules and the basal plane of graphene. Moreover, oxygen-containing moieties lead to electrostatic repulsion from phosphate ions and OH- moieties form hydrogen bonding to ascorbic acid [73]. Subsequently, it is suggested that the differentiation of hMSCs may be attributed to the affinity of rGO toward osteogenic differentiation-inducing factors such as cell essential ions and proteins. These observations, which could have general significance, demonstrate the potential of rGO-coated Ti substrates to promote the osteogenic differentiation of hMSCs.

## Conclusions

Herein, rGO NPs were coated to the surface of Ti substrate using the MDD method to evaluate the osteogenic differentiation-inducing effect of prepared rGO-Ti substrates. rGO-Ti substrates not only enhanced the proliferation of hMSCs but promoted osteogenic differentiation of hMSCs. These results are mainly attributed to the specific characteristics of rGO such as structural defects, electrical conductivity, residual oxygen-containing moieties, and hydrophilic nature that lead to enhanced cell adhesion, protein adsorption from serum, and cell-cell or cell–matrix signaling. These positive effects finally led to the spontaneous differentiation of hMSCs. From these results, it is proved that the rGO-Ti substrates have the potential for bone differentiation-inducing effect, therefore, promoting osseointegration with original tissue. In conclusion, it is suggested that the rGO-Ti substrates can be exploited to craft a range of strategies for the development of novel dental and orthopedic bone implants to accelerate bone regeneration because these graphene-coated materials have potentials to enhance osteogenesis.

## Data Availability

All data generated or analyzed during this study are included in this published article.

## Abbreviations

AFM: Atomic force microscopy
ALP: Alkaline phosphatase
ANOVA: Analysis of variance
ARS: Alizarin red S
ECM: Extracellular matrix
CCK-8: Cell counting kit-8
DMEM: Dulbecco’s modified Eagle’s Medium
GO: Graphene oxide
hMSCs: Human mesenchymal stem cells
MDD: Meniscus-dragging deposition
NPs: Nanoparticles
rGO: Reduced GO
SD: Standard deviation
Ti: Titanium
